# Comparisons of Chemical Profiles and Gastroprotective Effects of Citri Sarcodactylis Fructus Pre- and Poststeam Processing

**DOI:** 10.1155/2020/8491375

**Published:** 2020-09-23

**Authors:** Yinji Zhu, Qiuxia Zhang, Ming Gao, Hongfei Wang, Hui He, Jinyu Wang, Kang Chen

**Affiliations:** ^1^College of Chinese Medicine, Guangzhou University of Chinese Medicine, Guangzhou 510006, China; ^2^Guangzhou Regenerative Medicine and Health Guangdong Laboratory, Guangzhou 510005, China

## Abstract

**Background:**

Citri Sarcodactylis Fructus (CSF) is widely used as folk medicine in traditional Chinese medicine (TCM). The dried and steam-processed CSF (SCSF) has been employed for harmonizing the stomach over thousands of years under the guidelines of TCM theory. However, little is known about the differences in chemical compositions between CSF and SCSF. Moreover, the gastroprotective effects of CSF and SCSF on ethanol-induced gastric mucosal injuries in rats have yet to be investigated. Consequently, the present study aimed to investigate the chemical differences and gastroprotective effects of CSF and SCSF, providing some experimental framework for the development of CSF and SCSF.

**Methods:**

The chemical compositions of CSF and SCSF extracts were determined using an ultra-performance liquid chromatography-quadrupole time-of-flight mass spectrometer (UPLC-Q-TOF-MS), and their gastroprotective effects of different doses were assessed in rats with ethanol-induced gastric injuries on the levels of oxidative stress and inflammatory cytokines.

**Results:**

A total of 42 components were identified in CSF and SCSF, and most of them were flavonoids, limonoids, coumarins, and glycosides. There were no differences in the compositions between CSF and SCSF, but the relative contents of the components were different. Among them, nine screened compounds were considered as potential discriminating markers responsible for the differences between CSF and SCSF. Besides, pretreatments with CSF and SCSF markedly improved the gastric mucosal injuries in rats for their antioxidant and anti-inflammatory properties. And SCSF exhibited a better gastroprotective effect than CSF.

**Conclusion:**

The compositions of CSF were unchanged after steam-processing, while the relative contents of their components were changed. These changes may be the major reasons for the differentiation of their efficacies. In addition, CSF and SCSF could alleviate ethanol-induced gastric mucosal injury through the enhancement of antioxidant and anti-inflammatory activities. SCSF exhibited a better gastroprotective effect than CSF, which emphasized the necessity of steam processing.

## 1. Introduction

Citri Sarcodactylis Fructus is a qi-regulating TCM derived from the dried ripe fruit *Citrus medica* L. var. *sarcodactylis* Swingle. It is officially named as Fo Shou in China. It has been used in traditional and folk medicines for thousands of years to treat liver-qi stagnation, chest pain, stomach irritation, loss of appetite, vomiting, frequent cough, and excessive phlegm, by harmonizing stomach, relieving the depressed liver, and reducing dampness and phlegm (Chinese Pharmacopoeia, 2015). However, it has been reported that the crude CSF possesses the property of dryness, which is prone to deplete qi and injure yin as a result of long-term administration. Processing is capable of altering the chemical constituents of the Chinese medicinal material, which can change its TCM property for treating different syndromes [1]. Yet, the processing method of CSF has not been documented in the Chinese Pharmacopoeia, 2015.

Lingnan region, the southernmost part of China, has formed its own unique processing technologies to characterize a wide variety of TCM decoction pieces. Steam processing is often applied on CSF to reduce its dryness in this region. According to the Specification for Processing Traditional Chinese Medicine Pieces of Guangdong Province 1984, the Lingnan characteristic processing procedure of CSF was recorded as follows: remove impurities, steam for 2-3 hours, and then dry. To inherit the tradition of the local processing technique, we selected this method to process CSF. In modern clinical practice, SCSF has been applied for the treatment of chronic superficial gastritis, gastric cancer, gastric ulcer, and gastric neurosis. Phytochemical studies have shown that flavonoids, coumarins, and limonoids are the major bioactive compounds of CSF [2]. For example, limonin (limonoid) exerted a protective effect on hepatic toxicity by attenuating inflammation and reducing oxidative stress [3]. The flavonoid hesperidin exhibited gastric healing activity in the ulcerated mucosa by alleviating oxidative damage at sites of ulceration [4]. To comprehensively investigate the chemical information, it is necessary to determine the constituents of CSF and SCSF. Nowadays, UPLC-Q-TOF-MS has been widely used to characterize the structural constituents of TCM owing to its high resolution, excellent sensitivity, reproducibility, accuracy, and capability of generating abundant fragment information [5–7]. Thus, this technology was employed to identify the constituents of CSF and SCSF.

In present, little is known about the chemical differences between CSF and SCSF and their gastroprotective effects on ethanol-induced gastric mucosal injuries in rats. Therefore, the aims of this study were to distinguish the chemical variations between CSF and SCSF by UPLC-Q-TOF-MS coupled with multivariate statistical analysis. Besides, the gastroprotective efficacies of CSF and SCSF in rats with ethanol-induced gastric mucosal injury were investigated by assessing the levels of oxidative stress and inflammatory cytokines. And these will provide some experimental references for the development of CSF and SCSF.

## 2. Materials and Methods

### 2.1. Chemicals and Reagents

Reference standards, hesperidin, diosmin, and bergapten with purities greater than 98%, were obtained from National Institute for Food and Drug Control (Beijing, China). Ethanol was supplied by Damao Chemical Reagent Factory (Tianjin, China). High-performance liquid chromatography (HPLC) grade methanol was obtained from Merck (Darmstadt, Germany). HPLC grade formic acid was purchased from Sigma (St. Louis, USA). Ultrapure water was purified by a Milli-Q water purification system (Millipore, MA, USA). Lansoprazole (LSZ) tablets and Sanjiu Weitai granules (SWG) were purchased from Hunan Warrant Pharmaceutical Co., Ltd. (Hunan, China) and China Resources Sanjiu Medical and Pharmaceutical Co., Ltd. (Guangzhou, China), respectively. Glutathione (GSH), superoxide dismutase (SOD), and malondialdehyde (MDA) kits were supplied by Nanjing Jiancheng Bioengineering Co., Ltd. (Nanjing, China). Tumor necrosis factor-*α* (TNF-*α*), interleukin-2 (IL-2), interleukin-6 (IL-6), and interleukin-10 (IL-10) enzyme-linked immunosorbent assay (ELISA) kits were obtained from MultiSciences (Lianke) Biotech Co., Ltd. (Hangzhou, China). Pierce™ BCA protein assay kit was purchased from Thermo Fisher Scientific Inc. (MA, USA). All other chemicals and reagents were of analytical grade.

### 2.2. Plant Materials and Steam Processing

The details of samples are shown in Table 1. The decoction pieces of CSFs and SCSFs were obtained from Zisun Chinese Pharmaceutical Co., Ltd. (Guangzhou, China) and Lingnan Traditional Chinese Medicine Tablets Co., Ltd. (Foshan, China), which were authenticated as *Citrus medica* L. var. *sarcodactylis* Swingle by Prof. Kang Chen (College of Chinese Medicine, Guangzhou University of Chinese Medicine, Guangzhou, China) and deposited in our group's laboratory. For comparative analysis, 1 kg of each CSF except for **No.2** was immersed in 200 mL ddH_2_O in a closed container until softness, and then steamed in a perforated stainless steel steam boiler for 2.5 h at normal atmosphere. After drying overnight in an oven at 60°C, SCSF was obtained. The quality controls of CSFs and SCSFs are shown in Supplementary Materials (available here).

### 2.3. Preparation of Herbal Extracts for UPLC-Q-TOF-MS Analysis

All samples were cut into homogeneous thin slices. Each sliced sample of CSF and SCSF were accurately weighed (5 g) in triplicate and extracted twice with 100 and 75 mL of 95% ethanol, respectively, via the heat reflux extraction method for 1.5 h. The obtained extracts were combined, filtered, and then concentrated to 10 mg/mL stock solution. Subsequently, the stock solution was filtered through a 0.22 *μ*m Millipore membrane prior to UPLC-Q-TOF-MS analysis.

Reference compounds, hesperidin, diosmin, and bergapten, were accurately weighed and dissolved in 95% ethanol as individual standard stock solutions at the concentrations of 0.36, 0.41, and 0.49 mg/mL, respectively. A mixture consisting of 200 *μ*L aliquots of all solutions was labeled as the quality control (QC) sample at the concentration of 10 mg/mL. To monitor the reproducibility and reliability of the analytical system, the QC sample was run before and after the MS analysis of every 3 samples.

### 2.4. UPLC-Q-TOF-MS Conditions

Sample analysis was performed by UPLC-Q-TOF-MS using a triple TOF™ 5600^+^ mass spectrometer system (AB SCIEX, Foster City, CA) coupled with a Shimadzu UPLC system (Nexera, UHPLC LC-30A, Japan). This UPLC system contained a binary pump, an auto sampler, and a column oven. The samples were loaded onto a C18 column (1.9 *μ*m, 2.1 × 100 mm; Shimadzu), with a column temperature maintained at 30°C, a flow rate of 0.2 mL/min, and an injection volume of 4 *μ*L. Mobile phases A and B were methanol and 0.1% formic acid (v/v) in water, respectively. The UPLC elution conditions were optimized as follows: linear gradient from 95% to 86% B (0–6 min), 86% to 51.8% (6–12 min), 51.8% to 50% (12–20 min), 50% to 15% (20–23 min), and isocratic 15% B (23–26.7 min).

The optimal parameters of the MS/MS detector were as follows: ion spray voltage, 5500 V; ion source temperature, 550°C; declustering potential (DP), 110 V; and collision energy (CE), ±45 V. An electrospray ionization (ESI) source was operated in both positive and negative ion modes. The nebulizer gas (gas 1), heater gas (gas 2), and curtain gas (gas 3) were set to 55, 55, and 35 psi, respectively. MS data were collected in the full-time scan mode within a mass range of 50–1200 Da. Data acquisition was conducted using the PeakView Software TM V1.1 (AB SCIEX, Foster City, CA).

### 2.5. Preparation of Herbal Extracts for Animals

The sectioned samples were accurately weighed (50 g) and extracted twice with 1000 and 750 mL of 95% ethanol, respectively, via the heat reflux extraction method for 1.5 h. The obtained extracts were combined and then subjected to drying under reduced pressure and temperature using a rotary evaporator, thus producing viscous residue. The yields of CSF and SCSF were the same, 27.5%, by referring to the weight of the starting materials. They were collected and stored at −20°C until further experiments.

### 2.6. Animals

Forty-eight male Sprague–Dawley rats (180–220 g) were obtained from Animal Experimental of Guangzhou University of Chinese Medicine (license no. SCXK (YUE) 2013–0034). Rats were housed under controlled environmental conditions (uniform temperature 25°C, relative humidity 40–70%, 12 h light/12 h dark cycle with lights on at 8:00 am). Animals were allowed to acclimatize for 1 week prior to any experimental procedures and had free access to standard rat chow and water in raised mesh-bottom cages. All experimental protocols in this work were approved by the Animal Experimental Ethics Committee of Guangzhou University of Chinese Medicine (Guangzhou, China). Treatments of rats were performed in strict accordance with ARRIVE (Animal Research: Reporting of In Vivo Experiments) guidelines.

### 2.7. Treatment Procedures

After acclimatization, the rats were randomly divided into eight groups (*n* = 6 per group) using randomized blocking design (weight). The rats in normal and model groups received distilled water (10 mL/kg), those in prevention groups received LSZ 3.6 mg/kg or SWG 600 mg/kg, and those in high- and low-dose pretreatment groups received CSF (CSF-L, 0.6 g/kg; CSF-H, 2.4 g/kg) and SCSF (SCSF-L, 0.6 g/kg; SCSF-H, 2.4 g/kg), respectively, by gavage once daily for consecutive 4 days. The low dose in CSF and SCSF groups corresponds to half of the clinical equivalent dose of humans and the high dose to two times the clinical equivalent of the normal clinical oral dose. SWG, a positive control in TCM, is often used for the treatment of superficial gastritis and erosive gastritis, which consists of Evodiae Leptae Caulis Seu Cacumen (Sanchaku), Murrayae Folium et Cacumen (Jiulixiang), Zanthoxyli Radix (Liang Mian Zhen), Aucklandiae Radix (Muxiang), Scutellariae Radix (Huangqin), Poria (Fuling), Rehmanniae Radix (Dihuang), and Paeoniae Radix Alba (Baishao). Then, the rats were fasted for 24 h and treated as described above on the fifth day before ethanol-induced gastric injury. One hour later, all animals except those in the normal group received ethanol (5 mL/kg) via oral gavage to induce gastric mucosal injuries. The rats in the normal group received an equivalent volume of distilled water. All rats were anesthetized with a single dose of sodium pentobarbital (40 mg/kg i.p.) 1 h after ethanol administration and then sacrificed. Stomachs of rats were immediately removed, opened along the greater curvature, and gently rinsed with ice-cold physiological saline. The gross gastric mucosal injuries were visually observed, calculated, and photographed. After that, each stomach was quartered. One moiety was immersed in 4% paraformaldehyde for 48 h and then subjected to histopathological assessment. The remaining moieties were isolated, frozen in liquid nitrogen, and stored at −80°C for biochemical analyses.

### 2.8. Determination of Macroscopic Gastric Mucosal Injury

The ulcer index scores were measured with a ruler and calculated by a researcher blinded to experimental groups according to the five-point scale method [8]. The evaluation standards were as follows: 1 = spot erosion; 2 = erosion length <1 mm; 3 = erosion length 1∼2 mm; 4 = erosion length 2∼3 mm; and 5 = erosion length ≥3 mm; and the score was doubled if the erosion width was >1 mm. The partial scores were then summed to obtain the total ulcer injury score for each animal.

### 2.9. Histopathological Analysis

The gastric biopsy samples fixed in 4% paraformaldehyde were dehydrated, embedded in paraffin, and cut into 5 *μ*m-thick sections. The sections were then stained with hematoxylin and eosin (H&E) for histopathological assessment by a qualified observer.

### 2.10. Measurements of Oxidative Stress and Inflammatory Cytokines

The levels of GSH, SOD, and MDA were determined by commercial assay kits, while those of IL-2, IL-6, IL-10, and TNF-*α* were measured by ELISA assay kits. Briefly, the gastric tissues stored at −80°C were weighed, minced, and then homogenized with Tris-buffer (20 mM, pH 7.4) on ice using a homogenizer. After centrifuging at 3000 rpm for 15 min at 4°C, the supernatants were collected for measurement, and the total concentration of protein in the supernatants was determined using the BCA protein assay kit according to manufacturer's instructions. The results of SOD, GSH, and MDA were expressed as U/mgprot, *μ*mol/gprot, and nmol/gprot, respectively, while those of IL-2, IL-6, IL-10, and TNF-*α* were presented as pg/mg.

### 2.11. MS Data Processing and Statistical Analysis

The UPLC-Q-TOF-MS original data were preprocessed by PeakView and MakerView softwares (AB SCIEX, Foster City, CA). The identification of different compounds was performed by matching with reference standards and their accurate mass and fracture information with those reported in literature and commonly used online MS databases (ChemSpider, HMDB) with a mass tolerance of less than 5 ppm. SIMCA 14.0 software (Umetrics, Sweden) for multivariate analysis was introduced to establish supervised orthogonal projections to latent structures discriminant analysis (OPLS-DA) models for CSFs and SCSFs. Furthermore, based on the OPLS-DA model, discriminating compounds with conditioned variable importance in projection (VIP) were screened.

Animal data analysis was performed using SPSS software version 24.0 (Chicago, USA). Experimental values were presented as mean ± SD (standard deviation). Statistical analysis was conducted using one-way analysis of variance (ANOVA). *P* value <0.05 was considered statistically significant.

## 3. Results

### 3.1. UPLC-Q-TOF-MS Analysis

**Figure 1:**
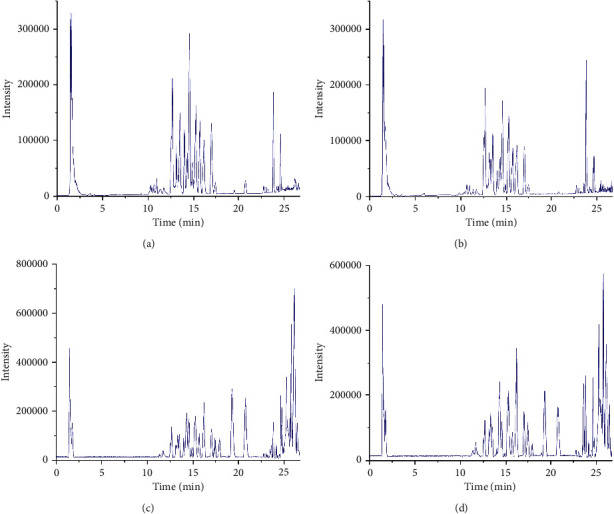
Base peak chromatograms of CSF and SCSF. The peaks of CSF (a) and SCSF (b) under the negative mode and those of CSF (c) and SCSF (d) under the positive mode.

UPLC-Q-TOF-MS analysis was conducted to determine the compositions of CSFs and SCSFs extracts. According to the chemical results shown in Table 2, a total of 42 compounds were detected through standard references and constituent structural information. Among them, 41 compounds were unequivocally identified, including flavonoids, limonoids, coumarins, glycosides, and other compounds. There was no significant difference in the compositions between CSF and SCSF, but the relative contents of the components were different (Figure 1). Specifically, the contents of 12 compounds were increased after steam processing, while those of 30 compounds were decreased. Among them, the content of glycosides was lower in SCSF than that in CSF. As for limonoids, the contents of nomilinic acid, obacunone, and limonin were higher after processing, while nomilin was the opposite. Furthermore, the contents of saccharides and amino acids were reduced after steam processing. These results suggest that the changes in chemical contents may be responsible for the variations between CSF and SCSF.

### 3.2. Multivariate Statistical Analysis

**Figure 2:**
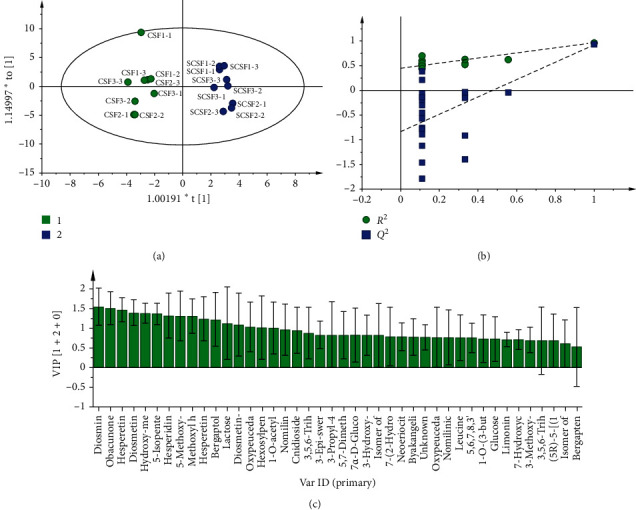
Multivariate statistical analysis of the chemical information of CSF and SCSF. OPLS-DA score plot (a), permutation test plot (b), and VIP plot (c).

OPLS-DA is a supervised model that can be used to determine the chemical variations between CSFs and SCSFs. A clear discrimination was achieved between CSF and SCSF (Figure 2(a)). The values of *R*^2^*Y* and *Q*^2^ were 0.972 and 0.935, respectively, indicating the good fitness and predictability of the OPLS-DA model. Furthermore, the permutation test revealed the good validity of the model (Figure 2(b)). Potential important chemical variables were obtained by filtering the compounds with VIP > 1.3. According to this criterion, there were nine compounds identified as potential distinguishing markers between CSFs and SCSFs (Figure 2(c)), including diosmin (**19**), obacunone (**15**), hesperetin (**34**), diosmetin (**36**), hydroxymethoxy coumarin (**16**), 5-isopentenyloxy-7-methoxycoumarin (**13**), 5-methoxy-8-hydroxypsoralen (**14**), hesperidin (**28**), and methoxy heptadecanoic acid (**37**).

### 3.3. Gastroprotective Effects of CSF and SCSF on Ethanol-Induced Gastric Mucosal Injury

**Figure 3:**
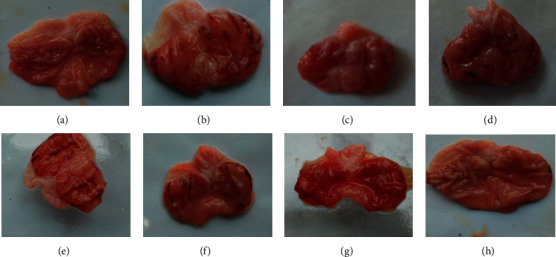
Effects of CSF and SCSF on the macroscopic appearance of gastric mucosa in rats with ethanol-induced gastric injury. (a) Normal group; (b) model group; (c) LSZ group; (d) SWG group; (e) CSF-L group; (f) CSF-H group; (g) SCSF-L group; and (h) SCSF-H group.

**Figure 4:**
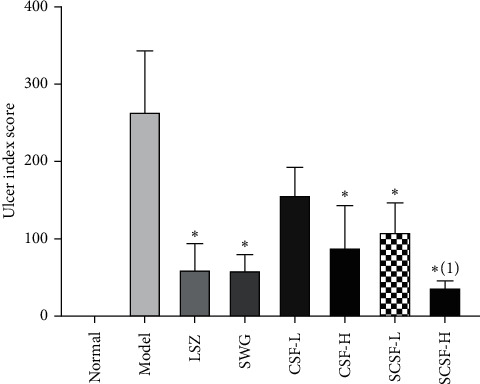
Effects of CSF and SCSF on the ulcer index score of gastric mucosa in rats with ethanol-induced gastric injury. The data are expressed as mean ± SD (*n* = 6). Note: ^*∗*^*P* < 0.05 vs. model group; ^(1)^*P* < 0.05 vs. CSF-H group.

As shown in Figure 3, the gastric mucosa of rats in the normal group was intact, and the gastric gland was visible. Intragastric administration of ethanol triggered the acute onset of hemorrhagic and necrotic mucosal lesions, along with the elongated and dark red band generally parallel to the long axis of the stomach and multifocal erosion. After pretreatments with CSF, SCSF, LSZ, and SWG, the gastric mucosal injuries were attenuated. Simultaneously, the ulcer index scores (Figure 4) were significantly declined in all pretreatment groups, except for the CSF-L group, by reducing the number and length of gastric ulceration (*P* < 0.05). As a well-known gastric mucosal protectant, LSZ can afford remarkable gastric protection. Notably, the ulcer index score of the SCSF-H group was lower than that of LSZ, indicating that SCSF exhibited better protective efficacy as LSZ on ethanol-induced gastric mucosal injuries. In addition, the SCSF-H group exhibited more significant gastroprotective effects than CSF-H on attenuating the ulcer index score (*P* < 0.05). This suggests that steam processing is necessary for CSF.

### 3.4. Histopathological Evaluations

**Figure 5:**
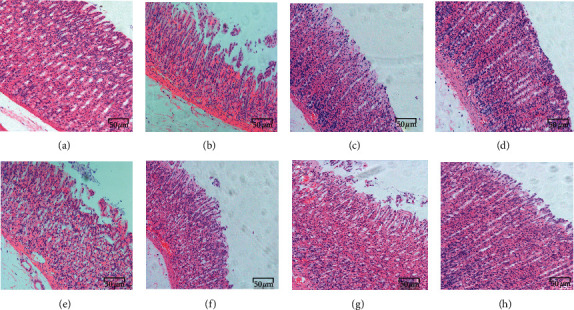
Effects of CSF and SCSF on the histopathological features of gastric mucosa in rats with ethanol-induced gastric injury rats. (a) Normal group; (b) model group; (c) LSZ group; (d) SWG group; (e) CSF-L group; (f) CSF-H group; (g) SCSF-L group; and (h) SCSF-H group. 400x magnification.

The histopathological data of gastric mucosa in rats with ethanol-induced gastric injury are depicted in Figure 5. In the normal group, the gastric mucosa of rats was intact, and the layers of the mucosa displayed clear boundaries (Figure 5(a)). On the contrary, the gastric mucosa of rats in the model group was seriously damaged, as evidenced by the disorganized structure of glandular, desquamation of epithelial cells, and infiltration of inflammatory cells (Figure 5(b)). However, the animals pretreated with CSF and SCSF had relatively enhanced protection with only mild disruption of the surface epithelium mucosa, especially in the high-dose SCSF group (Figure 5(h)).

### 3.5. Effects of CSF and SCSF on the Levels of GSH, SOD, and MDA

**Figure 6:**
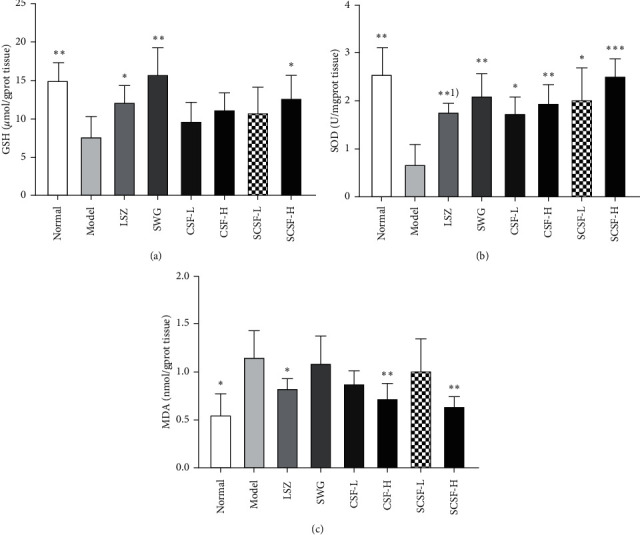
Effects of CSF and SCSF on the gastric mucosal levels of GSH (a), SOD (b), and MDA (c) in rats with ethanol-induced gastric injury. The data are expressed as mean ± SD (*n* = 6). Note: ^*∗*^*P* < 0.05, ^*∗∗*^*P* < 0.01, ^*∗∗∗*^*P* < 0.001 vs. model group; ^(1)^*P* < 0.01 vs. SCSF-H group.

As shown in Figure 6, ethanol-induced acute gastric mucosal injury resulted in the decreased levels of GSH and SOD and increased level of MDA. The levels of GSH were significantly elevated in LSZ and SCSF-H groups when compared to those in the model group (*P* < 0.05). All the pretreatments led to significant increases in the gastric levels of SOD relative to the model group (*P* < 0.05). In addition, the activity of SOD in SCSF-H group was significantly higher than that in the LSZ group (*P* < 0.001). Furthermore, the high-dose CSF and SCSF treatment groups and the LSZ group exhibited lower MDA levels compared with the model group (*P* < 0.01). These results indicate that CSF and SCSF can attenuate oxidative stress and maintain oxidant-antioxidant balance.

### 3.6. Effects of CSF and SCSF on the Levels of Proinflammatory Cytokines

**Figure 7:**
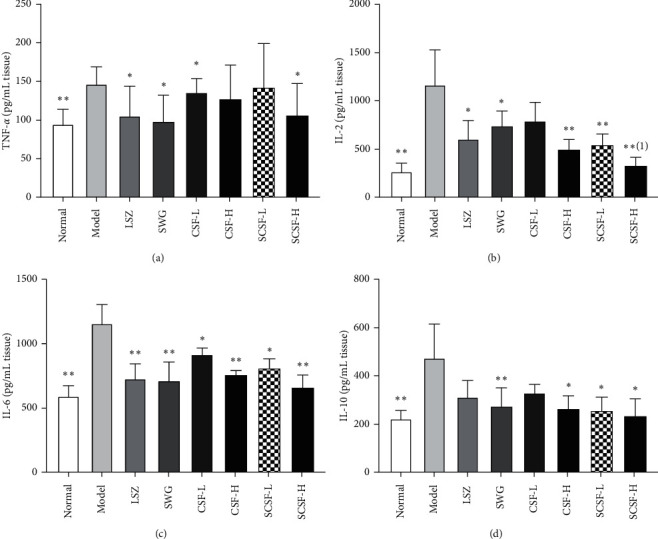
Effects of CSF and SCSF on the levels of inflammatory cytokines in rats with ethanol-induced gastric injury (*n* = 6). The data are expressed as mean ± SD (*n* = 6). Note: ^*∗*^*P* < 0.05, ^*∗∗*^*P* < 0.01 vs. model group; ^(1)^*P* < 0.05 vs. CSF-L group.

As shown in Figure 7, the gastric mucosal levels of proinflammatory cytokines, such as TNF-*α*, IL-2, IL-6, and IL-10, were significantly higher in the model group than in the normal group (*P* < 0.01). LSZ, SWG, and SCSF-H pretreatments markedly reduced the levels of TNF-*α*(*P* < 0.05) when compared to the model group, while no significant differences were observed in CSF-L, CSF-H, and SCSF-L pretreatment groups. Intragastric administration of CSF-H, SCSF-L, and SCSF-H could dramatically reduce the levels of IL-2 in rats with ethanol-induced gastric injury (*P* < 0.01). Specifically, the level of IL-2 in the SCSF-H group was significantly lower than that in the CSF-L group (*P* < 0.05), indicating that steam processing can enhance the gastroprotective effect of CSF. Moreover, the levels of IL-6 in all pretreatment groups were significantly decreased (*P* < 0.05) compared to those in the model group. Besides, the levels of IL-10 were markedly reduced in SWG, CSF-H, SCSF-L, and SCSF-H pretreatment groups (*P* < 0.05), while LSZ and CSF-L groups showed no significant differences when compared to the model group. These results are supportive of the gastroprotective effects of CSF and SCSF on gastric mucosa by attenuating the production of proinflammatory cytokines.

## 4. Discussion

According to our results of chemical composition analysis, both CSF and SCSF were mainly composed of flavonoids, limonoids, and coumarins. The flavonoids tended to generate [M − H]^−^ ions, while limonoids and coumarins were prone to generate [M + H]^+^. Therefore, UPLC-Q-TOF-MS was operated in both positive and negative ion modes to obtain more comprehensive information. The peak areas were changed between CSF and SCSF. The content of glycosides was decreased in SCSF compared to that in CSF, which may be attributed to the decomposition of glycosides during steam processing. Compared with CSF, the changes of limonoids, with higher nomilinic acid, obacunone, and limonin contents and lower nomilin content in SCSF, indicate that limonoids may be converted internally. In addition, saccharides and amino acids were reduced after steam processing, suggesting that the Maillard reaction may occur during this process. Furthermore, compounds **16** and **38** appeared at m/z 192.04 Da, while compounds **17** and **31** appeared at m/z 202.02 Da, with the same molecular weight. Their MS/MS fragment ions were almost the same because they had similar structures but different retention times. Therefore, we speculated that these compounds might be isomers, and further investigation is needed to distinguish them.

The chemical information of CSF and SCSF were analyzed by the OPLS-DA model to identify and distinguish their variabilities. In OPLS-DA analysis, the VIP value is the most commonly used index to evaluate the contribution of variables, and it is generally considered that the variables with a VIP > 1 are deemed statistically significant [9]. However, there are a large number of compounds at the threshold. Thus, these compounds were filtered by modifying the threshold values to VIP > 1.3. Then, nine compounds were identified as potential discriminating components between CSF and SCSF, mainly including flavonoids and their glycosides and coumarins. It suggests that these compounds are significantly changed during steam processing, and the changes of their contents may be the major reasons for the pharmacological differences between CSF and SCSF.

The gastric injury caused by alcoholism is a problem that raised worldwide concern. Alcohol would primarily destruct the gastric mucosa resulting in the depletion of mucus and bicarbonate accompanied by the enhancement of oxidative stress and inflammation followed by gastric injury [10, 11]. To model ethanol-induced gastric injuries, experiments on rats have been widely applied due to their similarities to human beings [12]. In our experiments, a single oral administration of ethanol could damage the gastric mucosa, leading to severe macroscopic and microscopic gastric injuries, which are characterized by adenoidal hyperemia, mucosal edema, point or line hemorrhage, and inflammatory cell infiltration. These results are in agreement with those reported in the literature [13]. However, pretreatments with CSF and SCSF ameliorated gastric injuries provoked by ethanol administration, especially in the SCSF-H group, indicating that CSF and SCSF can inhibit the development of gastric mucosal injury.

Ethanol-induced gastric injury is often accompanied by the production of reactive oxygen species (ROS), such as superoxide anion, hydrogen peroxide and hydroxyl radical, which in turn leads to cell death by interacting with a large number of molecular factors. Both enzymatic and nonenzymatic antioxidants, such as SOD and GSH, can scavenge these oxygen free radicals and protect against gastric tissue damage [14]. However, the excessive generation of ROS may enhance lipid peroxidation and deplete these antioxidant enzymes [15]. MDA, an end-product of lipid peroxidation, has been used to reflect the severity of oxidative stress [16]. Our findings showed that the level of GSH in the SCSF-H group was significantly higher than that in the model group. Moreover, the contents of SOD in pretreatment groups were significantly increased compared to the model group, and SCSF exerted better antioxidant activity than CSF. Additionally, the levels of MDA were significantly decreased in CSF-H and SCSF-H groups when compared to the model group. These results suggest that the gastroprotective effects of CSF and SCSF may be attributed to their antioxidant activities.

Cytokine networks contain a variety of cell damaging factors that lead to the development of gastric injury. The increased levels of several proinflammatory cytokines were observed in gastric tissue after ethanol administration. TNF-*α*, a potent stimulator of neutrophil infiltration, can activate and accumulate neutrophils around the injury sites, resulting in gastric microcirculatory disturbance and gastric damage. In addition, TNF-*α* can promote the secretion of interleukin-1, IL-6, and other cytokines to trigger inflammation [17]. IL-2 plays an important role in signal inhibition for the proper regulation of inflammatory response. Growing evidence has demonstrated that pleiotropic activity of IL-2, in the context of inflammation, is involved in proinflammatory and regulatory pathways and the interaction with other cytokines [18]. IL-6 is widely associated with inflammation, which mainly activates neutrophils and macrophages at the inflammatory sites. It can also induce oxidative stress and lysosomal enzymes, thus leading to gastric mucosal lesion [19]. IL-10 is mainly produced by macrophages and plays an important role in downregulating inflammatory cascade. It is responsible for suppressing the activation of macrophages and secretion of inflammatory cytokines such as TNF-*α*, IL-1*β*, and IL-6 in various types of tissues [20]. In the present study, pretreatments reduced the levels of TNF-*α*, IL-2, and IL-6, indicating their anti-inflammatory effects against ethanol-induced gastric injury. A distinct impact of the model group was observed for IL-10 production in gastric tissue, in which the levels of IL-10 in SCSF groups were almost normalized to basal level as compared to the normal group. Although unexpected, these results are consistent with those reported in another research [21]. It is not clear whether an increase in IL-10 production is associated with ethanol-induced gastric mucosal injury, but it is obvious that the inflammation cascade in the stomach may contain a mixed pattern of cytokines and immune regulatory events. In summary, our findings suggest that the gastroprotective efficacy of CSF and SCSF can also be attributed to their anti-inflammatory activities.

Because of the complex composition of CSF and SCSF, further investigations are needed to comprehensively attribute the observed differences in pharmacological effects to individual or several compounds.

## 5. Conclusions

Our findings indicated that there are no differences in chemical compositions between CSF and SCSF, but the contents of their compounds are different. In addition, the gastroprotective effects of CSF and SCSF against ethanol-induced gastric mucosal injury could be attributed to their antioxidant and anti-inflammatory activities, in which SCSF exhibit better gastroprotective efficacy than CSF. These experimental results encourage further studies on CSF and SCSF.

## Figures and Tables

**Table 1:** Sample information of 3 batches of CSFs.

No.	Origin	Batch number	Type
1	Zisun Chinese Pharmaceutical Co., Ltd.	171101	CSF
2	Zisun Chinese Pharmaceutical Co., Ltd.	171101	CSF and SCSF
3	Lingnan Traditional Chinese Medicine Tablets Co., Ltd.	1808001	CSF

**Table 2:** UPLC-Q-TOF-MS characterization data of CSF and SCSF under positive or negative ion modes.

No.	Identification	Formula	*T*_*R*_ (min)	*m*/*z*	Mode	Error (ppm)	MS^2^ fragments (m/z)	Trend
1	3-Hydroxy-2-methoxy-5,6-dimethyl-benzoic acid	C_10_H_12_O_4_	12.63	196.07	M+H	−3.4	151, 137, 118, 109	↑
2	7-(2-Hydroxyethoxy)-6-methoxy-coumarin	C_12_H_12_O_5_	13.86	236.06	M+H	−2.6	119, 133, 147, 149, 162, 177	↓
3	Hesperetin-5-O-glucoside	C_22_H_24_O_11_	14.38	464.13	M+H	−2.7	303, 285, 231, 219, 153, 145	↓
4	Oxypeucedanin hydrate	C_16_H_16_O_6_	16.79	304.09	M+H	−1.3	119, 131, 147, 159, 175, 203	↑
5	Limonin	C_26_H_30_O_8_	16.77	470.19	M+H	−1.5	161, 201, 213, 205, 277, 407, 425, 453	↑
6	Byakangelicol	C_17_H_16_O_6_	17.58	316.09	M+H	−0.9	117, 134, 175, 203, 218, 231, 230, 233	↑
7	5,7-Dimethoxycoumarin	C_11_H_10_O_4_	18.94	206.05	M+H	−0.9	107, 121, 135, 149, 164, 163, 192	↓
8	Bergapten	C_12_H_8_O_4_	20.15	216.04	M+H	−1.7	115, 118, 131, 146, 156, 174, 202, 217	↑
9	Oxypeucedanin	C_16_H_14_O_5_	22.89	286.08	M+H	−0.8	119, 131, 147, 159, 175, 202, 203	↓
10	3,5,6-Trihydroxy-3',4',7-trimethoxyflavone	C_18_H_16_O_8_	23.68	360.08	M+H	−0.5	183, 245, 285, 287, 315, 345	↑
11	Nomilinic acid	C_28_H_36_O_10_	23.67	532.23	M+H	−0.8	437, 341, 205, 161	↑
12	Nomilin	C_28_H_34_O_9_	20.43	514.22	M+H	−0.5	515, 469, 391, 307, 261, 205, 161, 95	↓
13	5-Isopentenyloxy-7-methoxycoumarin	C_15_H_16_O_4_	24.57	260.10	M+H	−2.3	199, 121, 117, 149, 175, 177, 205	↓
14	5-Methoxy-8-hydroxypsoralen	C_12_H_8_O_5_	24.82	232.03	M+H	−3.3	106, 134, 145, 161, 190, 218, 217	↓
15	Obacunone	C_26_H_30_O_7_	23.61	454.19	M+H	0.9	455, 179, 149, 189, 59	↑
16	Hydroxy‐methoxy coumarin	C_10_H_8_O_4_	25.30	192.04	M+H	−2.8	105, 122, 133, 150, 149, 161, 178, 178	↓
17	Bergaptol	C_11_H_6_O_4_	25.17	202.02	M+H	1.6	117, 117, 145	↓
18	Leucine	C_6_H_13_NO_2_	1.79	131.09	M+H	−0.1	86, 69, 56	↓
19	1-O-(3-butenyl)-6-O-*α*-L-arabinosyl-*β*-D-glucopyranoside	C_15_H_26_O_10_	11.11	366.15	M-H	0.9	101, 125, 221, 282	↑
20	3-Methoxy-4-O-*β*-D-glucosyl benzoate	C_16_H_22_O_9_	12.64	358.12	M-H	1.3	151, 195	↑
21	5,6,7,8,3'4'-Hexamethoxyflavone-3-O-(3-hydroxy-5-methoxy-3-methyl-5-oxopentanoyl)-*β*-D- glucopyranoside	C_34_H_42_O_18_	12.64	738.23	M-H	0.8	217, 357, 379, 737	↓
22	Cnidioside-B	C_18_H_22_O_10_	12.88	398.12	M-H	0.0	235, 217, 217, 201, 176, 161	↓
23	7-Hydroxycoumarin	C_9_H_6_O_3_	12.93	162.03	M-H	2.2	105, 161, 89, 65	↓
24	Diosmetin-6,8-di-C-*β*-D-glucopyranoside	C_28_H_32_O_16_	12.96	624.16	M-H	2.5	312, 383, 503	↓
25	Neoeriocitrin	C_27_H_32_O_15_	13.26	596.17	M-H	0.9	459, 287, 151, 135	↓
26	3-Epi-swertiajaposide C	C_17_H_24_O_10_	13.42	388.13	M-H	0.7	166, 181, 225, 207, 151	↓
27	Hexosylpentose-flavonoid glycoside	C_27_H_30_O_15_	14.25	594.15	M-H	1.0	285, 593	↓
28	Hesperidin	C_28_H_34_O_15_	14.39	610.19	M-H	1.8	286, 301	↓
29	Diosmin	C_28_H_32_O_15_	15.36	608.17	M-H	1.5	284, 299	↓
30	1-O-Acetyl-3-O-[-1-oxo-3(4-hydroxy-3-methoxyphenyl)-2-propene]*β*-D-fructofuranosyl-2,3,6-triacetate-*α*-D-glucopyranoside	C_30_H_38_O_18_	16.62	686.20	M-H	0.9	191, 235, 279, 397, 439, 541	↓
31	Isomer of peak 17	C_11_H_6_O_4_	16.77	202.02	M-H	1.6	117, 117, 145	↓
32	3-Propyl-4-methyl-5,7-diethoxycoumarin	C_17_H_18_O_8_	16.81	350.10	M-H	0.8	117, 201	↓
33	Unknown	C_32_H_42_O_17_	17.24	698.24	M-H	1.4	195, 194, 357, 697	↑
34	Hesperetin	C_16_H_14_O_6_	19.11	302.07	M-H	−0.1	301, 285, 201, 164, 136, 108	↓
35	3,5,6-Trihydroxy-4',7-dimethoxyflavones	C_17_H_14_O_7_	24.28	330.07	M-H	−0.4	127, 171, 255, 271	↓
36	Diosmetin	C_16_H_12_O_6_	23.28	300.06	M-H	−0.5	107, 255, 256	↓
37	Methoxy heptadecanoic acid	C_18_H_32_O_3_	26.18	296.23	M-H	−0.3	114, 115, 158	↓
38	Isomer of peak 16	C_10_H_8_O_4_	13.09	192.04	M-H	−2.8	105, 149, 161, 178	↑
39	Glucose	C_6_H_12_O_6_	1.51	180.06	M-H	2.1	89, 85, 71, 59	↓
40	Lactose	C_12_H_22_O_11_	1.54	342.11	M-H	−0.7	341, 221, 179, 149, 119, 89, 59	↓
41	(5R)-5-[(1R)-1,2-Dihydroxyethyl]-*β*-D-lyxopyranosyl-(1->6)-*β*-D-glucopyranosyl-(1->2)-*α*-D-Glucopyranose	C_19_H_34_O_17_	1.55	534.18	M-H	0.9	191, 127, 85	↓
42	7*α*-D-Glucopyranosyloxy-2,3,4,5,6-pentahydroxyheptanoic acid	C_13_H_24_O_13_	1.52	388.12	M-H	−0.6	341, 221, 179, 149, 119, 89, 59	↓

## Data Availability

The data used to support the findings of this study are included within the article.

## Supplementary Materials

Supplementary Materials The contents of hesperidin in CSFs (Table S1) met the standard (not less than 0.030%) in the Pharmacopoeia, 2015, while the contents in SCSFs had not been documented.
